# Compliance with central venous catheter infection prevention practices after intervention with simulation

**DOI:** 10.1590/0034-7167-2022-0574

**Published:** 2023-10-09

**Authors:** Thayane Gusmão Pires de Oliveira, Juliana de Oliveira Marcatto, Allana dos Reis Corrêa, Luciano Marques dos Santos, Patrícia Kuerten Rocha, Delma Aurélia da Silva Simão, Bruna Figueiredo Manzo

**Affiliations:** I Universidade Federal de Minas Gerais. Belo Horizonte, Minas Gerais, Brazil; II Universidade Estadual de Feira de Santana. Feira de Santana, Bahia, Brazil; III Universidade Federal de Santa Catarina. Florianópolis, Santa Catarina, Brazil

**Keywords:** Health Education, Infant, Nursing Care, Intensive Care Units, Catheter-Related Infections., Educación en Salud, Posmaduro, Atención de Enfermería, Unidades de Cuidado Intensivo Neonatal, Infecciones Relacionadas con Catéteres., Educação em Saúde, Recém-Nascido, Cuidados de Enfermagem, Unidades de Terapia Intensiva Neonatal, Infecções Relacionadas a Cateter.

## Abstract

**Objectives:**

to assess the effect of an educational intervention based on clinical simulation on nursing professionals’ compliance with practices to prevent peripherally inserted central venous catheter-associated primary bloodstream infections in a Neonatal Intensive Care Unit.

**Methods:**

a quasi-experimental study, with preand post-intervention assessment with a single group. The population consisted of 41 nursing professionals, with 31 observations being made before and after the intervention. Analyzes were performed using descriptive statistics and the McNemar non-parametric test. A significance level of 5% was adopted.

**Results:**

after the intervention, there was an increase in compliance with prevention practices of surgical antisepsis and professional hand hygiene, skin antisepsis with chlorhexidine, waiting for the time of the effect of alcoholic chlorhexidine and compliance with the sterile technique.

**Conclusions:**

the educational intervention showed an effect on increasing compliance with catheter-associated infection prevention practices.

## RESUMO

**Objetivos:**

avaliar o efeito de uma intervenção educativa pautada em simulação clínica na adesão de profissionais de enfermagem às práticas de prevenção de infecções primárias de corrente sanguínea associadas ao cateter venoso central de inserção periférica em Unidade de Terapia Intensiva Neonatal.

**Métodos:**

estudo quase-experimental, com avaliação pré e pós-intervenção com um grupo único. A população foi de 41 profissionais de enfermagem, sendo realizadas 31 observações antes e após intervenção. As análises foram por meio de estatística descritiva e pelo Teste Não Paramétrico de McNemar. Adotou-se um nível de significância de 5%.

**Resultados:**

após a intervenção, houve aumento da adesão às práticas de prevenção de antissepsia cirúrgica e higiene das mãos do profissional, antissepsia da pele com clorexidina, espera do tempo do efeito da clorexidina alcoólica e cumprimento da técnica estéril.

**Conclusões:**

a intervenção educativa mostrou efeito no aumento da adesão às práticas de prevenção da infecção associadas ao cateter.

## RESUMEN

**Objetivos:**

evaluar el efecto de una intervención educativa basada en simulación clínica sobre la adherencia de profesionales de enfermería a prácticas de prevención de infecciones primarias del torrente sanguíneo asociadas a catéteres venosos centrales de inserción periférica en una Unidad de Cuidados Intensivos Neonatales.

**Métodos:**

estudio cuasiexperimental, con evaluación pre y postintervención con un solo grupo. La población estuvo constituida por 41 profesionales de enfermería, realizándose 31 observaciones antes y después de la intervención. Los análisis se realizaron utilizando estadística descriptiva y la prueba no paramétrica de McNemar. Se adoptó un nivel de significancia del 5%.

**Resultados:**

después de la intervención, hubo aumento en la adherencia a las prácticas de prevención de antisepsia quirúrgica e higiene profesional de manos, antisepsia cutánea con clorhexidina, espera del tiempo del efecto de la clorhexidina alcohólica y cumplimiento de la técnica estéril.

**Conclusiones:**

la intervención educativa mostró un efecto en el aumento de la adherencia a las prácticas de prevención de infecciones asociadas al catéter.

## INTRODUCTION

The involvement of healthcare-associated infections (HAI) and nosocomial bacteremia in newborns are directly associated with mechanical ventilation duration, birth weight and the need to use devices for the survival of these children, such as central venous catheter (CVC)^(1)^. Knowledge about infection prevention is of paramount importance, as the growing infectious dissemination in bloodstream can lead to high costs in patient care, increased length of hospital stay and a higher morbidity and mortality rate^(1-2)^.

Among intravenous devices, peripherally inserted central catheters (PICC) are most commonly used to ensure durability of central venous access and can be inserted at the bedside by trained professionals^(3)^. The insertion of this device is carried out by trained doctors and nurses, and the nurse in charge is responsible for training the nursing team in relation to device care^(3)^. It is also noteworthy that PICC insertions in the Neonatal Intensive Care Unit (NICU) are mostly performed by nurses^(4)^ and it is up to these professionals to comply with the guidelines regarding their handling as well as to recognize early signs and symptoms related to mechanical, thrombotic and infectious complications^(3)^.

PICCs are used for newborns (NB) who need administration of antibiotic therapy, infusion of hyperosmolar solutions or solutions with non-physiological pH for 6 or more days^(5)^. Complications involving infectious phlebitis, catheter obstructions, bleeding, among others, can occur, and the longer the device remains, the greater the chances of acquiring HAIs^(5)^. Thus, direct care with device preservation and weighting are necessary^(5)^.

Studies warn that CVC use has increased rates of central venous catheter-associated bloodstream infection (CVC-BSI) in NICUs^(6-8)^. Descriptive study with a quantitative approach, which contemplated the density of CVC-BSI in Brazil, showed that, from 2015 to 2019, there was an increase in infections of this nature, which represented an average of 7.55 infections per 1,000 catheter-days^(7)^. While in Europe, the incidence rates of sepsis associated with CVC accounted for 20% of this type of event and have a mortality rate of around 30%^(8)^. In the United States, an average of 250,000 CVC-BSI notifications occur per year, a mortality rate of 25% and treatment costs reaching up to 60,000 dollars for each event^(9)^.

In order to avoid CVC-BSI, preventive measures have been proposed worldwide^(10)^. Among them, the Guidelines for the Prevention of Intravascular Catheter-Related Infections recommended using the bundle, which consists of a set of care for certain risk situations, such as actions that prevent adverse events (AE) arising from using devices invasive^(10)^. The care recommended by PICC insertion and maintenance bundles are: hand hygiene; hub disinfection before being accessed; maximum barrier precautions; hygiene techniques when changing dressings; insertion site selection using aseptic techniques with chlorhexidine; waiting for the action time of chlorhexidine; and daily assessment of the need for the PICC to remain^(11)^.

A study carried out in a NICU in Australia, which used the PICC care checklist (bundle), identified significant reductions between the pre-intervention and intervention groups. The numbers show a drop in rates from 8.8 per 1,000 catheter days to 4.9 per 1,000 catheter days^(12)^. A study that analyzed bundle care showed a reduction in AE from 32% to 19.6% and from 37.3% to 15.2% in low birth weight NBs^(13)^. Thus, educational measures are able to reduce peripherally inserted CVC-BSI, however they require that the elements that make up the bundle be fulfilled by all professionals involved^(14)^.

The development of protocols, guidelines and training aimed at preventing peripherally inserted CVC-BSI are essential to guide and qualify practice, however the theory is not always transposed into the NICU team’s daily work^(3,15)^.

The set of good practices provided for in CVC-BSI prevention bundle can be complied with by the health team through educational actions. Among them, we can mention clinical simulation use, which is a dynamic experiential learning strategy that meets the needs of professionals to improve in simulated real contexts, repeatable and that adds theoretical and practical aspects to its execution, offering security to those involved^(16)^. However, until now there is a scarcity of studies that assess the effect of educational interventions based on clinical simulations on compliance with CVC-BSI prevention practices in neonatology, which is the focus of this study. The hypothesis of this study is that the educational intervention with clinical simulation use influences the increase in compliance with peripheral insertion CVC-BSI prevention practices by the nursing team in a NICU.

This study is justified by obtaining elements that can support more effective educational practices in the search for greater compliance with CVC-BSI prevention measures and, consequently, patient safety (PS) in NBs using PICCs.

## OBJECTIVES

To assess the effect of an educational intervention based on clinical simulation on the compliance of nursing professionals with peripherally inserted CVC-BSI prevention practices in NICUs.

## METHODS

### Ethical aspects

The study was conducted in accordance with national and international ethics guidelines and approved by the Research Ethics Committee of the *Universidade Federal de Minas Gerais* and the hospital involved. The Informed Consent Form was obtained from all individuals involved in the study in writing.

### Study design, period and place

This is a quasi-experimental, preand post-intervention study with a single comparison group. The SQUIRE 2.0 instrument from the EQUATOR network was used to guide this methodology.

The setting consisted of a NICU with 25 beds in a teaching and research hospital, aimed at the Brazilian Health System (SUS - *Sistema Único de Saúde*) patients, which provides comprehensive care for women and NBs, in Belo Horizonte, MG, Brazil.

Data collection took place between January 2019 and February 2020, denominated in pre-intervention, intervention and post-intervention period respectively. It was decided to carry out the post-test 1 (one) month after the end of the intervention.

### Study population; inclusion and exclusion criteria

The professionals eligible for the study were nurses qualified to insert the PICC and nursing technicians at the NICU, who are responsible for maintaining the PICC.

Nurses qualified for PICC insertion and day and night shift nursing technicians who were linked to the care of newborns using a PICC, with more than 6 months of experience in the NICU, were included. Professionals who, during the preor post-intervention observation, requested to interrupt their participation in the research or who had not participated in 100% of the intervention were excluded.

Among the eligible professionals, the study sample consisted of 10 nurses who provide direct patient care and who have qualifications in PICC insertion and 31 nursing technicians from the NICU, totaling 41 professionals.

### Study protocol

The research protocol was applied in three moments: pre-intervention, intervention and after intervention.

### Pre-intervention phase

In the first phase of this study, the research proposal was presented to the target population and the risks, benefits, secrecy, voluntary participation and the Informed Consent Form (ICF) were explained. Of the total number of potential participants, six nursing technicians and two nurses refused to participate in the research. Furthermore, before starting data collection, all nursing professionals were invited to individual or group meetings so that the purpose and relevance of the study could be presented. At that moment, the ICF was signed.

This phase consisted of collecting data on the participants and observing the practice of inserting and/or manipulating the PICC before the intervention.

The variables listed to describe the characteristics of the nursing professionals responsible for inserting and/or handling the PICC were age, time passed since graduation, time working in the NICU, weekly workload and type of job.

The variables related to infection prevention practices during PICC insertion before and after the intervention were: surgical antisepsis technique of the hands of the professional in charge of PICC during insertion; Personal Protective Equipment (PPE) use by the assistant (nursing technician); PPE use by the professionals in charge of PICC during insertion; skin antisepsis with 2% chlorhexidine degermant; rubbing chlorhexidine in alcoholic solution ≥ 0.5% for 30 seconds; waiting for the time of action of alcoholic chlorhexidine of 2 minutes; hand hygiene after insertion and whether there was a breach of sterile technique during catheter insertion.

To assesss the practice of preventing infection during device maintenance, the following variables were used: hand hygiene before and after handling the catheter; antisepsis with 70% ethyl alcohol solution for at least 15 seconds before opening connections; checking the validity of venous line connections; and daily review of catheter need with immediate removal of unnecessary catheters.

Direct observation of PICC insertion and handling procedures by nursing professionals was carried out from March to June 2019, with the aim of providing an analysis of the care practices developed during catheter insertion and handling. A type of instrument (checklist) based on the CVC-BSI prevention actions and on the bundle was used, comprising two parts: verification items during catheter insertion and items that should be observed during PICC handling. This instrument was assessed by 6 experts in the area of safety in neonatology and infection control before its use, with the need for minor adjustments regarding assessment description. The observations were made by previously trained researchers and occurred during the day and night until 9 pm.

Direct observation was carried out by scientific initiation students known to the team, but who did not work in the service, in a natural way so that it did not influence the behavior patterns regarding the procedure. These students participated in a previous training where two researchers of the study, through discussions of CVC-BSI prevention measures manuals of the Center for Disease Control and Prevention, World Health Organization (WHO) and Brazilian National Health Regulatory Agency (ANVISA - *Agência Nacional de Vigilância Sanitária*), previously read, followed by simulations, debriefing and clarification of doubts regarding care practices that would be observed.

Direct observation was used as a data collection method, as this is characterized by being a meticulously planned action with a view to meeting pre-established criteria. Nurses were assessed at the PICC insertion at least 2 times for each professional, totaling 31 observations before and after the intervention. Nursing technicians were assessed regarding the process of handling the device, totaling 31 observations before and after the intervention. It is noteworthy that nurses were assessed more than once because there is a smaller number of professionals qualified to be part of the PICC.

### Intervention phase

In the second phase, an educational intervention was carried out through clinical simulation, which had the PICC bundle in the NICU as its guiding axis. This intervention took place over five days lasting 20 to 25 minutes, in different shifts (morning, afternoon and evening), with groups of 4 to 5 people, between September 30 and October 4, 2019. The coordinator divided the professionals into groups according to shift schedule and beds. During this period, the unit had an additional nursing professional on the scale to support care during training. The intervention took place in a room attached to the NICU and used a bed in a neonatal unit with all the relevant equipment in a controlled environment and with a low fidelity dummy which are static simulators, and simulated patients, which are actors trained to act and reproduce user behaviors in different situations and health care establishments. A case was presented of a NB admitted to the NICU who would need a PICC procedure and we would have to proceed with the insertion and handling of this device. In view of this, 2 nurses were chosen, voluntarily, to perform the insertion procedure, in addition to two other nursing technicians, to provide care during the device maintenance. After simulation, debriefing was carried out with the presence of a moderator and participants, redoing the correct action when necessary and emphasizing safe practices in PICC-related infection prevention. It is noteworthy that a checklist similar to the data collection instrument was used by a moderator to monitor participants’ practical performance.

### Post-intervention phase

Participants’ observation period was 30 days after the educational intervention, from November 21, 2019 to February 28, 2020. The same instruments used to assess the practice of PICC insertion and handling procedures by nursing professionals in first stage were used in the period after the intervention by the same researchers.

### Analysis of results, and statistics

The information was transferred to an Excel spreadsheet and analyzed using the IBM Statistical Package for the Social Sciences (SPSS) program, version 20.0. Qualitative variables were described using absolute and relative sequences. For the numerical values and for the inferential analysis to verify the nursing team’s compliance with prevention measures in PICC insertion and handling before and after the educational intervention, McNemar’s non-parametric test was used according to the Shapiro-Wilk test application. A significance level of 5% was adopted. The checklist items or variables were marked by means of two alternatives, being “yes” when practice was performed correctly and “no” incorrectly.

## RESULTS

Of the 10 participating nurses, all were female; 72% were between 25 and 35 years old; 80% had graduated between 10 and 20 years ago; and 78% had worked in the NICU for more than 10 years. Of the 31 nursing technicians, all were female; 82% were between 25 and 35 years old; 84% had graduated between 5 and 15 years ago; and 80% with time working in the NICU between 10 and 15 years. The workload of most participants is 30 hours, and their type of employment was public servants.

Comparing the percentage of compliance in relation to PICC infection prevention practices during the device insertion by nurses before and after the educational intervention, a significant increase in compliance was observed regarding the change in attitude regarding professionals’ antisepsis during insertion, skin antisepsis with chlorhexidine, waiting for alcoholic time, hand hygiene after insertion and if there was a break in the sterile technique (Table 1).

**Table 1:** Comparisons of compliance percentage in relation to peripherally inserted central catheter infection prevention practices during device insertion by nurses before and after the educational intervention, Belo Horizonte, Minas Gerais, Brazil, 2022 (N=31)

Variables	Intervention		*p* value ^a^
	Before n(%)	After n(%)	
Surgical antisepsis of professionals’ hands during insertion			0.004^a^
Yes	21(67.7)	30(96.8)	
No	10(32.3)	1(3.2)	
PPE use by the assistant			-
Yes	31(100.0)	31(100.0)	
No	0(0.00)	0(0.00)	
PPE use by professionals during insertion			-
Yes	31(100.0)	31(100.0)	
No	0(0.00)	0(0.00)	
Skin antisepsis with 2% degerming chlorhexidine followed by alcohol ≥ 0.5% for 30 seconds			<0.001^a^
Yes	7(19.4)	25(80.6)	
No	24(77.4)	6(19.4)	
Waiting time for action of alcoholic chlorhexidine for 2 minutes			<0.001^a^
Yes	1(3.2)	24(77.4)	
No	30(96.8)	7(22.6)	
Hand hygiene after insertion			<0.001^a^
Yes	19(61.3)	30(96.8)	
No	12(38.7)	1(3.2)	
Was there a break in the sterile technique?			0.012^a^
Yes	10(32.3)	1(3.2)	
No	21(67.7)	30(96.8)	

the McNemar Test; PPE - Personal Protective Equipment.

Regarding the percentages of compliance with the PICC infection prevention actions during the two moments of device maintenance by nursing technicians before and after the educational intervention, there was a significant increase in compliance regarding the change of attitude towards hygiene actions of hands before handling, antisepsis before opening connections and hand hygiene after handling (Table 2).

**Table 2:** Nursing technicians’ compliance with measures to prevent bloodstream infection during peripherally inserted central catheter handling in the preand post-intervention period, Belo Horizonte, Minas Gerais, Brazil, 2022, (N=31)

Variables	Intervention		*p* value ^a^
	Before n(%)	After n(%)	
Hand hygiene before handling			<0.001^a^
Yes	15(48.4)	29(93.5)	
No	16(51.6)	2(6.5)	
Antisepsis with 70% ethyl alcohol solution for at least 15 seconds before opening connections			<0.001^a^
Yes	24(6.5)	26(83.9)	
No	5(93.5)	5(16.1)	
Check validity of venous line connections			-
Yes	31(100.0)	31(100.0)	
No	0(0.00)	0(0.00)	
Daily review of the need for catheter replacement			-
Yes	31(100.0)	31(100.0)	
No	0(0.00)	0(0.00)	
Hand hygiene after handling			-
Yes	8(25.8)	26(83.9)	
No	23(74.2)	5(16.1)	

the McNemar test.

## DISCUSSION

The present study had the objective of assessing the effect of an educational intervention based on clinical simulation principles in relation to the preventive actions of peripherally inserted CVC-BSI during the moments of insertion and maintenance of this device by the nursing team in the NICU. The results showed the positive effect of the educational intervention in relation to compliance with peripherally inserted CVC-BSI prevention practices by the nursing team in a NICU.

This study revealed that most prevention actions had an effect on compliance after the intervention. In this regard, a survey points out that using educational technologies in health training optimizes the educational process and method, in addition to increasing the acquisition of technical-scientific knowledge, resulting from professionals’ experiences, providing the health team with systematization and security in care^(17)^. In the simulation environment, the professional has the opportunity to discover gaps in their knowledge, develop new cognitive foundations and improve their intervention skills^(17)^. Moreover, using simulation improves knowledge about physiology, pathophysiology, diagnostic accuracy, treatment and team role^(18)^.

Simulation-based training uses artificial representation of real-world processes to enable hands-on learning^(19)^. In addition, education is a key component of infection prevention efforts, encompassing lectures, videos and fact sheets with some hands-on opportunities^(19)^. Using simulation has grown in the health area, and its applicability is a complement to traditional teaching methods in preventing infections, PS and reducing costs with incidents^(19)^.

Considering the increase in compliance after the educational intervention observed in the results of this study, it can be said that it is consistent with other educational interventions based on infection prevention^(20-21)^. It is believed that the results obtained contributed to the agreement of the nursing team in seeking alternatives in peripherally inserted CVC-BSI prevention, since NBs admitted to the NICU are susceptible to these infection.

As an estimate of preventive actions, the systematization of care provides strategies to minimize CVC-BSI^(11)^. Thus, educational intervention based on simulation is a way to improve the processes and outcomes of patient care. Some studies reinforce that using simulation in the health area promotes some benefits, as the ability of participants to develop opportunities to examine decisions to be made, promote communication and problem-solving skills, increase interest in building improvements, and make increased learning possible^(20-21)^.

The institution of the Brazilian National Policy on Permanent Education in Health (PNEPS - *Política Nacional de Educação Permanente em Saúde*) provides for the execution of educational actions based on the reality of services that induce transformations in work processes and reflections on professional practice, through active methodologies, such as simulation^(22)^. In this sense, the National League for Nursing also emphasizes that using the simulated scenario is an opportunity for practice, learning, assessment, testing or even to gain understanding of systems or human actions^(21)^. Another study also shows that simulation can favor the development of psychomotor and social skills, communication, creativity, teamwork and collaboration, critical thinking and clinical learning^(23)^.

In the literature, hand hygiene (HH) has been prioritized by the WHO since 2004, as an important and recognized measure in the fight against HAIs^(24)^. Considering the importance of the HH technique, a Brazilian study showed an increase in nursing team compliance in neonatal wards with regard to HH before and after handling the CVC, being 80% and 90%, respectively^(25)^. These data converge with the current study as a statistically significant difference was observed after the intervention. In assessing nursing professionals’ compliance after the intervention, it is observed that they started to adopt surgical hand antisepsis actions before catheter insertion, after device insertion and HH before and after handling the device.

The data obtained in this study are also similar to those of a research that sought to assess HH compliance among health professionals in an Intensive Care Unit (ICU) before and after the educational intervention^(26)^. The results proved to be efficient regarding global HH compliance, obtaining a significant increase from 30.9% to 69.5% after intervention^(26)^. Although this study worked with an adult population, the findings showed that offering educational actions, such as simulated scenarios, is effective in contributing to increased compliance with activities based on patient care.

One of the likely explanations for the improvement in nursing professionals’ compliance with CVC-BSI prevention actions, in this study, refers to the preparation methodologies and knowledge of the PICC technique during insertion and handling. Studies indicate that interventions carried out through the training of health professionals within multidisciplinary teams, for assistance to critical patients in NICUs in hospitals in developing countries, such as El Salvador, Mexico, Philippines and Tunisia, have proved to be effective^(27)^. The mean density of peripherally inserted CVC-BSI, which was 21.4 episodes/1,000 days of CVC, was reduced to 9.7 episodes/1,000 days of PICC^(27)^. In Brazil, a quasi-experimental study, which used the training of professionals through training as an intervention strategy, reveals that the mean density of peripherally inserted CVC-BSI in the neonatal population was reduced by 31.03 episodes/1,000 PICC days to 2.9^(28)^.

These findings converge with our results, which demonstrate that the simulated scenario also as an educational tool had an influence with regard to the possibilities of reducing the rates of peripherally inserted CVC-BSI, since professionals’ compliance with HH practices was satisfactory.

It is known that all nursing professionals involved in inserting the PICC must use the maximum precautionary barrier, which consists of using a long-sleeved apron, gloves, an expanded sterile field, a mask covering the nose and mouth, and a cap, which must cover all the hair^(29)^.

The inappropriate use of elements that make up PPE could be a harbinger of cross-infection, increased unnecessary costs for the health institution and, as a consequence, the decrease in HH^(30)^. An integrative literature review, which sought to highlight the main nursing care when handling invasive devices such as the PICC in an adult ICU, showed that only 33% of 18 studies analyzed mentioned PPE use during the procedure^(31)^. Despite this finding, most available evidence considers this intervention to be important, as it reduces microbiological contamination of the central catheter insertion site and, therefore, the risk of bloodstream infection^(1)^.

Given the above, the results showed that PPE use by assistants and professionals during insertion is consolidated among professionals, not interfering with the results before and after the intervention. The fact that the rates of compliance with PPE use did not show changes is justified by the veracity of the Hospital Infection Control Service (HICS) team of the study unit already developing in-service education actions as well as the monitoring and dissemination (feedbacks) of health professionals’ compliance with various PS components. Although the responses to this variable have not been modified, it is important to emphasize that the continuity of reinforcement actions regarding PPE use in favor of reducing peripherally inserted CVC-BSI must be maintained.

Another important point refers to the antisepsis of the site to be punctured with 2% degerming chlorhexidine, followed by alcohol ≥ 0.5%, for 30 seconds, performed through unidirectional movements. This technique is an important measure to reduce the risk of peripherally inserted CVC-BSI, since alcoholic chlorhexidine is more effective in reducing the rates of microbial colonization on NB’s skin when compared to other antiseptics^(32)^. Its action is effective against Gram-positive and Gram-negative microorganisms, yeasts and some viruses, even in the presence of organic substances on the skin. In addition, it has an important residual action that prolongs its suppressive activity against microorganisms^(32)^.

Applying skin preparation with alcoholic chlorhexidine for asepsis is part of the bundle of measures aimed at installing and maintaining the PICC^(1)^. Adequate skin preparation with using alcoholic chlorhexidine ≥ 0.5% for 30 seconds, performed through unidirectional movements, ensures the effectiveness and safety of PICC insertion site preparation and maintenance^(33)^. In this sense, ANVISA recommends that, before proceeding with the puncture, one should wait for the time of action of alcoholic chlorhexidine^(1)^. Using this theoretical framework in the intervention allowed us to verify that, in relation to our study, in the pre-intervention period, only 3.2% of professionals waited for the product to dry on the patient’s skin before inserting the catheter. Above all, after the educational action, there was a significant increase of 74.2% in nursing professionals’ compliance, demonstrating that the study obtained influence and safety for health professionals’ compliance in the PICC insertion site preparation and maintenance. This is reflected in the action taken by the simulated scenario, in which professionals were able to learn and practice correctly when performing the antisepsis of the site to be punctured.

One of the aspects that it was possible to perceive so that nursing professionals’ acceptance in relation to the adequate chlorhexidine use and the recommended waiting time would obtain better results, the emphasis that the incorrect use of antisepsis of the site to be punctured can lead to AE with regard to the premature skin, when the educational action through simulation took place. Thus, this emphasis is consistent, considering that a Brazilian study, which verified compliance with the PICC insertion bundle in neonatal and pediatric units, showed that, among the sample of 31 (52.5%) insertions performed, there was a technical error during degermation on patients’ skin^(31)^. The inappropriate use of alcoholic chlorhexidine was 37.3%, and 27.1% of procedures had contamination^(31)^. This mentioned study also converges with a descriptive American study, which showed that 22 (41.5%) who used chlorhexidine gluconate inappropriately resulted in the occurrence of burns (54.5%), redness (2.9%), dermatitis (4.5%) and other skin irritations (31.8%)^(32)^.

It is expressed that the ideal care at the time of insertion are essential measures in PICC-related infection prevention. However, risks are still present during the device’s permanence time, since the central lines at the time of catheter handling can be accessed 30 to 50 times during the day^(34)^.

With regard to catheter handling, ANVISA recommends that professionals who manipulate the device have specific qualifications and training, aiming at the safety of the care process^(1)^. When there is a suspicion of bloodstream infection, early PICC removal is performed through needleless connectors (NC). Its operation occurs through coupling to the catheter’s hub (cannon), where drug administration, solution infusions, blood sample withdrawal and connection of infusion equipment are performed^(35)^. Infections are correlated with the presence of microorganisms on the tip of the catheter and in blood culture^(35)^. A randomized clinical trial with 300 NC demonstrated that 51% of devices were contaminated by microorganisms from patients’ skin^(36)^. A study published by the Royal College of Nursing showed reductions in infection rates due to using bundles as a focus to prevent infections when handling catheters^(37)^. Actions such as HH, aseptic technique before opening catheter connections, changing the dressing and checking the catheter and rubbing the hub before handling the devices were performed^(37)^. This reference reinforces the actions used at the time of simulation, which contributed to nursing technicians’ compliance with this practice, being 83.9% after the intervention. Likewise, the practice used shows that the need for training of the health team that performs PICC insertion, maintenance and removal can be fundamental to reduce the risks of AE for patients^(38)^.

This fact is in line with a descriptive study with a quantitative approach, of the systematic observation type, which aimed to identify the care strategies adopted by nursing professionals in PICC handling in children and NB. It mentions that rigorous rubbing for 5-15 seconds with 70% ethyl alcohol provides a significant reduction in PICC-related infections^(25)^. During routine handlings, it is considered a barrier strategy against the colonization of microorganisms, given that the opening of connections constitutes a gateway to the infusion circuit^(25)^. For this reason, research considers catheter hub colonization to be the main cause of 50% of catheter-related infections after catheter insertion^(25)^.

In short, it was noticed that the simulated scenario as an educational tool helps in the compliance of health professionals responsible for PICC insertion. Furthermore, it enables the process of learning and practicing the knowledge applied to device insertion and handling techniques.

Through responses to compliance with intervention practices, the continuity of professional training and permanent education of the nursing team in the NICU is essential. In this way, the risks are minimized, NB safety is guaranteed and the assistance is qualified^(39)^.

### Study limitations

The main limitations of this study include the fact that the research was carried out in only one public institution, restricting the extrapolation or comparison of results. Also noteworthy is the reduced number of observations, since the research time was limited, thus not allowing to analyze the effect of the intervention in the long term. Therefore, what is recommended is that future studies investigate the long-term effect of the educational action on compliance with peripherally inserted CVC-BSI prevention measures, in order to monitor the sustainability of nursing team’s changes in behavior and attitudes.

### Contributions to nursing

The findings of this study indicate that educational intervention based on simulation can help health professionals and teachers to rethink teaching strategies aimed at compliance at satisfactory levels to infection prevention measures related to the PICC. In addition, the results of this study favor evidence for clinical practice, which can help in the translation of knowledge, as it is a replicable educational strategy, in addition to encouraging researchers and professionals to develop research on this topic, especially in neonatal units, in search of a safer practice for patients.

## CONCLUSIONS

This study provided evidence that the educational intervention based on simulation positively influences nursing professionals’ compliance with peripherally inserted CVC-BSI prevention actions, both in the device insertion and maintenance. Moreover, it contributes to the dissemination of knowledge through using educational strategies that may favor scientific evidence use in clinical practice by the nursing team, in search of a more qualified and safe care.
