# ERRATUM

**DOI:** 10.1590/0034-7167.20257801e02

**Published:** 2025-02-03

In the article “Compliance with central venous catheter infection prevention practices after intervention with simulation”, with DOI number: https://doi.org/10.1590/0034-7167-2022-0574, published in Revista Brasileira de Enfermagem, 2023;76(4):e20220574, on page 7:

Included:

**FUNDING**

Fundação de Amparo à pesquisa do Estado de Minas Gerais (FAPEMIG) - projeto APQ-02531-17.

