# Supplementary material for: ERRATUM
Source: Rev Bras Enferm. 2025 Feb 3;78(1):e2025n1e02. doi: 10.1590/0034-7167.20257801e02 (PMC11793164; doi:10.1590/0034-7167.20257801e02)
Supplement: Related texts

# Correction: ERRATA

**DOI:** 10.1590/0034-7167.20257801e02pt

No artigo “Adesão às práticas de prevenção de infecção de cateter venoso central após intervenção com simulação”, com número DOI: https://doi.org/10.1590/0034-7167-2022-0574pt, publicado no periódico Revista Brasileira de Enfermagem, 2023;76(4):e20220574, na página 8:

Inclui-se:

**FOMENTO**

Fundação de Amparo à pesquisa do Estado de Minas Gerais (FAPEMIG) - projeto APQ-02531-17.
